# Determinants of household’s waste disposal practices and willingness to participate in reducing the flow of plastics into the ocean: Evidence from coastal city of Lagos Nigeria

**DOI:** 10.1371/journal.pone.0267739

**Published:** 2022-04-28

**Authors:** Nnaemeka Andegbe Chukwuone, Ebele Chinelo Amaechina, Innocent Abanum Ifelunini

**Affiliations:** 1 Department of Agricultural Economics, Resource and Environmental Policy Research Centre, University of Nigeria, Nsukka, Nigeria; 2 Department of Economics, Resource and Environmental Policy Research Centre, University of Nigeria, Nsukka, Nigeria; Tsinghua University, CHINA

## Abstract

Marine plastic pollution is a critical environmental challenge facing policymakers globally. To reduce marine plastic pollution by engaging the people, this study estimated the determinants of waste disposal approach by households, their willingness to participate in road gutters/drainage channels cleanup program and the number of man-days they are willing to contribute. The study used a total of 600 households drawn from 30 enumeration areas. A semi-structured questionnaire was employed in data collection. Means, percentages, multinomial logit model and Heckman selection model were employed in data analysis. The study found that most (67.42 percent) of the households in the coastal city of Lagos engage in illegal waste disposal. Some variables, household size, involvement in previous community cleanup activities, receipt of waste management information, payment of waste management fee, and having a dumpster in a locality, significantly reduce the likelihood of illegal waste disposal. The study also found that most (75.50 percent) of the households were willing to clean up road gutters/drainage channels; however, most (83.20 percent) were only willing to contribute one man-day (eight hours) in a week. Gender and previous participation in voluntary service significantly influenced both households’ willingness to participate and the number of the man-days they are willing to contribute. Women are more likely to participate and contribute man-days to the activity. Education, household size and amount paid as waste management fee significantly reduced the number of man-days households are willing to contribute. In contrast, the provision of information on waste management significantly increased the number of days they are likely to participate. The study recommended providing waste management information and dumpsters to reduce illegal waste disposal, mobilizing citizens, especially women, the less educated and low waste fee-paying households, through well-packaged information about plastic pollution.

## 1 Introduction

Marine plastic pollution is one of the major environmental issues threatening aquatic life, and its management is now considered a critical environmental priority [1]. Plastics found in the ocean, about 80 percent from land-based and mismanaged waste material, enter through inland waterways, wastewater outflows, and transport by wind or tides [2]. Out of 275 million metric tons of plastic waste generated in 2010 by 192 coastal countries, about 4.8 to 12.7 million metric tons (MMT) entered the ocean [2]. Nigeria ranked 9th out of 20 coastal countries with 0.13–0.34MMT/year of plastic marine debris in 2010 [2]. Poor waste disposal and management is a primary environmental concern as only 20-30percent of over 32 million tons of waste generated in Nigeria annually are collected [3]. This problem is of particular concern in Lagos, where over 10,000 tons of waste are generated daily by a population of over 20 million residents, with an average generation capacity of 0.5kg per capita per day [3]. Given a coastal city with limited waste management infrastructure and poor waste management, a large percentage of these wastes, 15percent of which are plastics [4], end up in road gutters/drainage channels, canals, waterways, lagoons, then the ocean. Therefore, to reduce the flow of plastics into the ocean, this study estimates the determinants of households’ willingness to participate in a cleanup of road gutters/ drainage channels program in the coastal city of Lagos, Nigeria, before plastic wastes enter the water bodies.

Marine plastic pollution resulting mainly from land-based sources contributed between 4.8 and 12.7 million tons in 2010 and is expected to double by 2025 if adequate measures are not taken to limit it [2]. Many plastics in the oceans are from mismanaged and illegal disposal practices [5]. These deposits often originate from households, construction, packaging and coastal tourism [6]. Due to poor waste management and disposal practices, plastic wastes enter the oceans through different inland water bodies. Besides land-based sources of plastic waste, marine plastic pollution also results from fishing especially discarded or abandoned fishing gear, with a global contribution of 640,000 tons of marine debris [7], shipping and other maritime activities, for example, aquaculture [8].

The impact of marine plastics pollution on marine life and human livelihoods is enormous. Plastics impact wildlife in the oceans through ingestion, entanglement, bioaccumulation and distortions in the ecosystem integrity [9]. Through entanglement and damage, plastics can reduce the productivity and efficiency of commercial fisheries and aquaculture [10]; and thus, impact the food security of 19 percent of the global population that fisheries constitute 20 percent of their food intake [11]. Although it is difficult to attach a specific value to the loss of ecosystem services related to marine plastic pollution, [12] postulated a reduction of 1–5 percent of marine ecosystem services due to the stock of marine plastics in oceans in 2011. This reduction rate equates to an annual loss of benefits generated from marine ecosystem services amounting to $500–$2500 billion. Research evidence shows that coupled with the impacts of climate change and overfishing, the fishing and aquaculture industry’s productivity, profitability, security, and viability are vulnerable to the impacts on marine plastic pollution [12]. Since fish is a crucial source of micronutrients, a decline in fish catch and productivity can exacerbate the micronutrient (zinc, iron and vitamin A) deficiency of 850 million people globally [11].

Countries have applied different policy instruments in order to deal with the problem of marine plastic pollution. Policy instruments have targeted plastic production, consumption and disposal. Policy instruments that have been used include price-based instruments, (e.g. increasing the prices or imposing taxes on plastic products); regulation instruments, (e.g. bans); rights-based instruments, (e.g. waste-based billing); and behavioral instruments (engaging the people), for example, education, information and cleanup campaigns [5,13]. In Africa, commonly used policy instruments by some countries to reduce the production and consumption of single-use plastics are taxes and bans [8]. Some countries in Africa, namely, Mauritania, Ghana, Kenya, Ethiopia, Côte d′ivoire, Mali, Malawi, Mauritius, Rwanda, Senegal, Tanzania and Uganda, have imposed different levels of bans on the use of plastic bags and some other single-use plastics while both Cameroon and South Africa have imposed taxes [8]. In Nigeria, although a National Policy on Plastic Waste Management was launched in 2020 [14], a country-wide ban or tax on the use of single-use plastics does not exist. The national policy’s stipulated ban of single-use on the go plastics such as plastic bags, cutlery, Styrofoam, and Straws is expected to come into effect in 2025. The national assembly passed a plastic bag probation bill in 2019, which prohibits plastic bags’ use, manufacture, and importation [15]. However, the bill was not signed into law.

Despite the use of bans and taxes and other policy initiatives in Africa, Sub-Saharan Africa (SSA) accounts for 9 percent of mismanaged plastic waste globally [16], even as the policy instruments have produced mixed results [17]. For example, while banning of plastic bags recorded some success in Rwanda, it was less successful in Kenya and Uganda [18] For Nigeria, over 850,000 tons of plastic waste are mismanaged yearly, with over 130,000 tons ending in water bodies [19]. Furthermore, there is no consensus regarding the environmental benefit and costs of banning single-use plastics. Some researchers argued that plastics have serious impact on the environment, for example, their climate change effects [20], and effect on aquatic life, especially due to risk of ingestion and entanglement [21], costing 13 billion USD in damage to the marine ecosystem [22] and should be banned in place of alternatives. Others, on the other hand, argued that alternatives to plastics like paper and glass make commensurate if or even higher impact on the environment due to depletion of forests as a result of paper production and high energy consumption required to manufacture other products, for example, glass and will equally lead to a tripling of greenhouse gas emission [23,24]. However, proponents of banning and non-banning of single use plastics agree on the need to have better waste management to limit the inflow of plastics into waterways and the oceans [25].

Thus, given that regulatory and price based policy instruments are still inefficient in dealing with marine plastic pollution in SSA and many developing countries [2,6], especially because of poor enforcement capacity [26], the lack of consensus on the benefits and cost of regulatory instruments, there is a need to explore additional alternatives, for example, behavioral instruments, to enhance plastic waste management and reduce the flow of plastics into the oceans. Besides, a mixture of policy instruments is recommended in managing a hydra-headed problem like marine plastic pollution. This is important for Nigeria, where marine plastic pollution is a critical environmental problem, and little or no policy measures have been taken to tackle the problem. Behavioral instruments, including education, outreach and community cleanup campaigns [13], tackle consumption and disposal of single-use plastics. Clean-up campaigns involve community members in the cleanup of plastic wastes, especially on beaches, to prevent them from entering the ocean and thus make the people become custodians of their environment. Some community cleanup campaigns include the international coastal cleanup [27] and keep America beautiful [28]. Also, the UN Environment in 2017 launched the clean seas campaign on marine litter that aims to address the root causes of marine plastic pollution with a five-year strategy that includes educating and engaging citizens, among others [29]. Research evidence [13] shows that behavioral instruments, for example, investment in campaigns, resulted in a more significant reduction of plastic pollution than policies relating to single-use plastic ban and taxes. Also [30], in a study on barriers and success factors to adopting sustainable municipal solid waste management in Nigeria, found that people’s engagement, especially through sustained public education regarding waste prevention and reuse, had a significant impact on the adoption of sustainable solid waste management. Although Lagos State practiced a three-hour monthly sanitation exercise on the last Saturday of every month and with movement restriction during the exercise some years ago, the government stopped the practice due to litigations and court rulings against movement restriction [31].

Therefore, to effectively engage people in dealing with marine plastic pollution, there is a need to understand their waste disposal approach, determine their willingness to participate and socioeconomic and other factors that can support people’s engagement. Policy instruments are more effective when the target population’s characteristics are considered in their design than not [32]. Although there have been previous studies on the effect of behavioral instruments on the reduction in the use of single-use plastics and marine plastic pollution, for example [13,33]; and that of [34] on the factors that influence participation of people in no plastic bag campaign; and [35,36] on the social cost of marine litter along European and China coasts, studies on the determinants of participation of people in cleanup campaigns to reduce marine plastic pollution are lacking. As noted earlier, littering and poor waste management in Lagos result in dumping in road gutters and drainage channels from where plastic wastes find their way to water bodies. Thus, the questions are: what are the determinants of household waste disposal approaches used by households? will households be willing to participate in the cleanup of road gutters/drainage channels program to limit the flow of plastic into the ocean? What factors influence their willingness to participate and contribute man-days of labor? Therefore, the study estimates Lagos households waste disposal approaches, their willingness to participate, and the number of man-days/hours they are willing to contribute to clean up road gutters/drainage channels to limit the flow of plastics into the ocean.

The rest of the paper is structured as follows: section two is on conceptual framework and literature review, section three focuses on the methodology, section four is result and discussion, and section five concludes.

## 2 Conceptual framework and literature review

**Fig 1:**
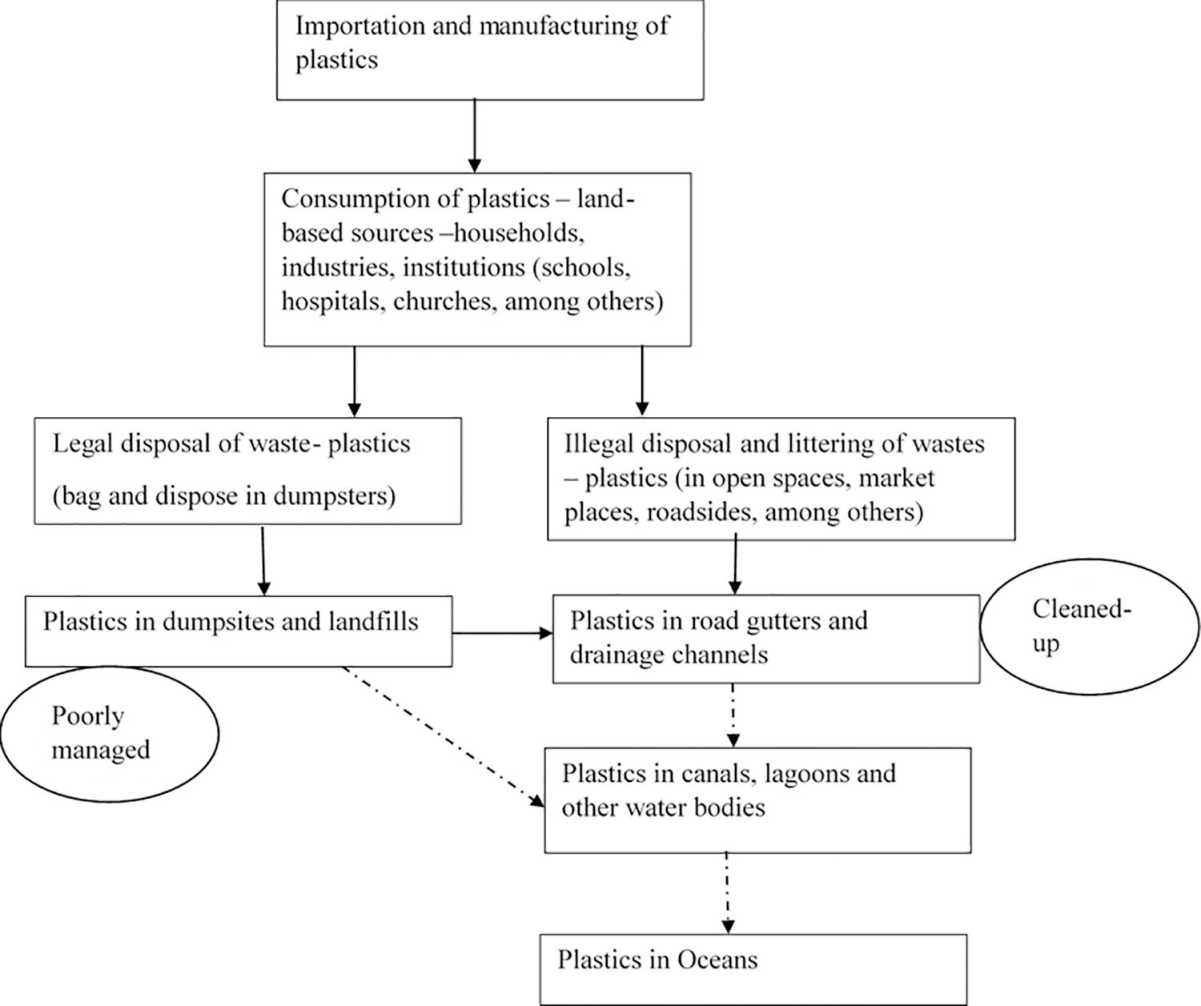
Conceptual framework showing the pathway of generation of plastics and their flow into the oceans.

In line with the conceptual framework (Fig 1), plastics pass through three stages of manufacture/importation, consumption and disposal before they end up in the ocean [5]. The total global plastic production has continued to grow annually from 225 million metric tons in 2004, reaching 288 million metric tons in 2012 and 311 metric tons in 2014 [2,37,38]. In Nigeria, plastic manufacturing started in the 1960s with about 50 companies in the sector. However, this grew to over 3000 companies manufacturing plastic products in 2013 with a capacity of over 100,000 tons of plastics annually [19]. Besides manufacturing, plastics are also heavily imported into Nigeria. About 20 million tons of primary plastics and plastic products were imported into Nigeria between 1996 and 2017, making the country the second-largest importer of plastic products in Africa, accounting for 17percent of Africa’s total consumption [19].

Plastics are consumed by households, industries, institutions (schools, hospitals, churches etc.). Single-use plastic products popularly used in Nigeria include plastic bags, especially polyethene (cellophane), popularly used in wrapping consumables, takeaway food packs, cups, straws, and spoons. However, plastic sachet bags for packaging water, often called "pure water", are the most widespread [39–42]. After a single use, these plastic products are either properly disposed of or littered and dumped in open places, roadsides, market places, among others. Properly disposed ones are collected by municipal waste collectors and disposed of in dumpsites and landfills. In Lagos, some dumpsites and landfills are managed by Lagos State Waste Management Authority (LAWMA) [4]. The illegally disposed and littered ones are blown by wind or stormwater into road gutters and drainage channels through which they end up in canals, lagoons, other water bodies, and the ocean. Limiting illegal waste, especially plastic waste disposal, would limit their entry into the oceans, hence to need to find a way to limit illegal disposal. Also, given poor management, some of the plastic wastes at the dumpsites are dispersed by wind and found in drainage channels and waterways, ending up in the ocean. However, cleaning up road gutters and drainage channels before the onset of rains, as indicated in Fig 1, will reduce the flow of plastics into waterways and oceans. Hence this study estimates the factors that influence the willingness of households to participate in the cleanup of road gutters and drainage channels to reduce the flow of plastics into the ocean.

Previous studies have determined factors that generally influence households’ participation in beach cleanup schemes and waste management. [35] found that gender, experience, familiarity with a particular beach, income level, and membership of an environmental protection organization influenced participation in beach cleanup schemes. Specifically, they found that female visitors, especially older ones, are more likely to participate and give up more days than males, especially younger males. Also, they found that higher-income respondents and members of an environmental protection organization are more likely to participate than lower-income and non-members. On the other hand [36], found that higher-income visitors were less likely to participate in a beach cleanup program in China’s Zhejiang province. [43] found that social perception, health and hygiene, concern about the environment, economic status, and lifestyle are the primary social determinants of public attitudes regarding waste management especially recycling. A study in Tanzania [44], found that the number of years of education, household income, peer group influence, and incentives significantly increased the likelihood of households participating in a waste separation for reducing, reuse, and recycling program. On the other hand, they also found that gender and income significantly reduce the likelihood of waste separation by households with men and those with high income less likely to separate solid wastes. A study in South Africa [45] found that being married, monthly income, willingness to pay for waste disposal, paying for waste disposal, race (white, Indian, colored), the existence of waste recycling programs and facilities positively and statistical significantly influenced households’ involvement in waste separation for recycling. In addition, a study in Uganda [46] found that household size, the income of household head, ownership of land/plot, and concern for the environment significantly increased the likelihood of household involvement in waste separation. At the same time, gender significantly reduced the likelihood of involvement in waste separation, with men being less involved in waste separation than women. On household’s solid waste disposal behavior [47], found that household educational level and local economic level positively and significantly influenced rural residents’ solid waste disposal behavior. On the other hand, they found that village layout and distance between residence and garbage collection facilities negatively and significantly influenced their solid waste disposal behavior [48], in a study on determinants of people participation in waste separation in Nigeria, found that the location of households impacted the way they feel about participation in solid waste separation activities as those in rural areas indicated greater willingness to participate than those in urban areas.

## 3 Methodology

### 3.1 Study area

**Fig 2:**
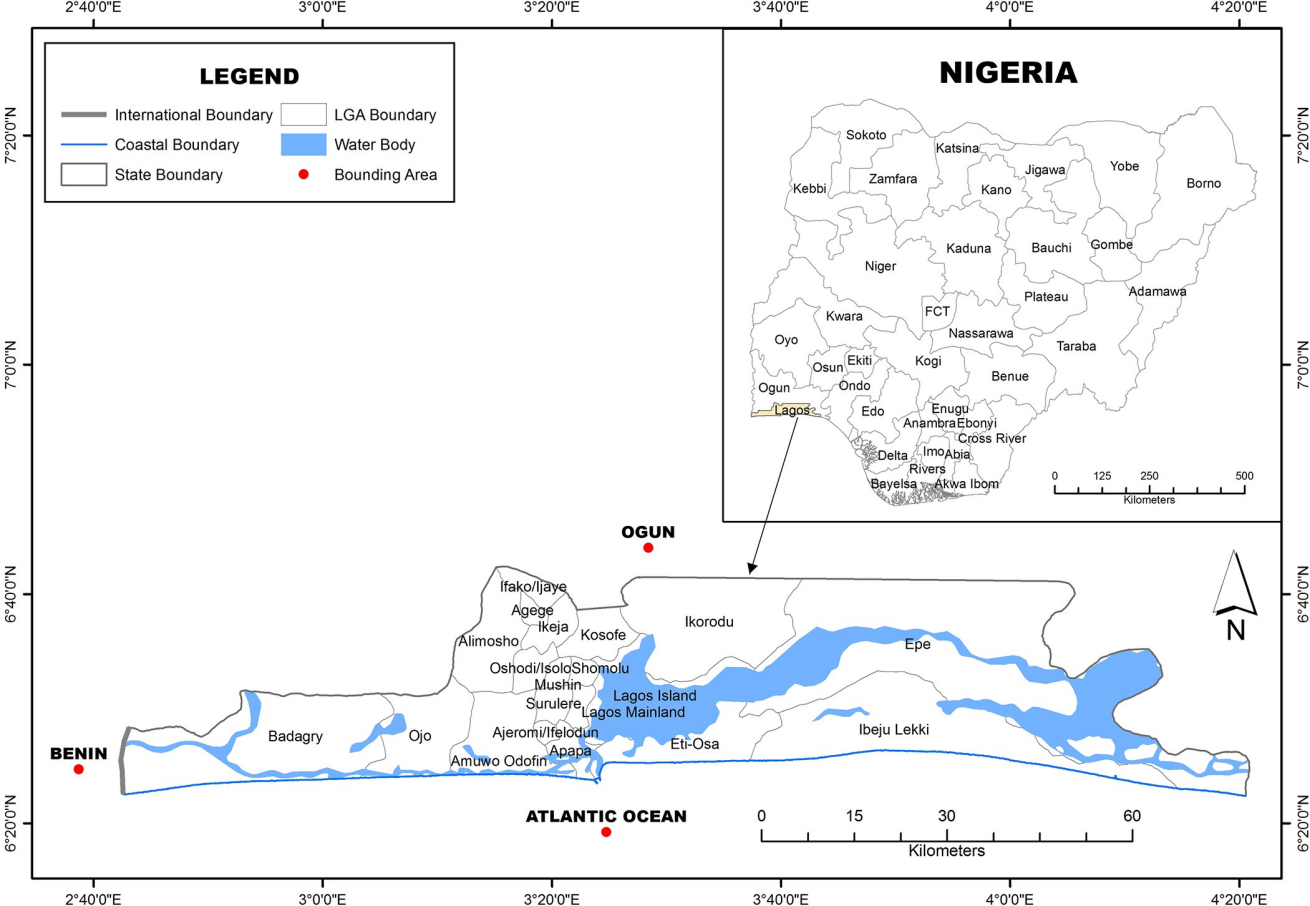
Map of Lagos with that of Nigeria inset. **Source:** Produced by the authors from Landsat Image Data obtained from United States Geological Survey (USGS) database at www.earthexplorer.usgs.gov.

The study area was Lagos State, Nigeria, a coastal State with 9,013,534 inhabitants in 2006 [49] and an annual growth rate of 3.2percent [3] estimated that over 10,000 tons of solid waste are generated in Lagos daily. Given poor waste management in Lagos, a large chunk of the waste end up in road gutters/drainage channels and are moved by stormwater into waterways, canals, lagoons and then the Atlantic Ocean. Fig 2 shows the map of Lagos with that of Nigeria inset.

### 3.2 Sampling procedure and sample size

To ensure a representative sample of households, household selection involved sampling of households from the Enumeration Areas (EA) used by the National Bureau of Statistics (NBS). EA is a cluster of housing units as delineated by the National Population Commission. A two-stage sampling process was applied in the selection of households for the study. First, we randomly sampled a total of 30 EAs for the study. From each EA, twenty (20) households were randomly sampled, giving a total of 600 households for the study.

### 3.3 Data collection

Data collection involved the administration of a semi-structured questionnaire with tablets using the "Survey Solutions" software [50]. The questionnaire consists of a preliminary section and three main parts. The preliminary section collected information about the local government area, enumeration area and global positioning system (GPS), the sector (whether urban or rural), and household identification. Also, in the preliminary section, the respondents were asked to indicate their consent to be interviewed. Those that did not give consent were not interviewed. Given that it was a survey and not human experimentation and we obtained the respondents’ consent, the study did not require further approval from our university’s Human Experimentation Ethics Committee (HEEC). The first part generated data on the respondents’ perception of single-use plastics and their environmental problems. In the second part, the respondents provided information regarding their willingness to participate in a program for the cleanup of wastes in road gutters/drainage channels before the onset of the rainy season and the number of man-days or hours they were willing to volunteer in a week. The third part focused on the socioeconomic attributes of the respondents. Well trained enumerators were used in data collection. Actual data collection lasted for seven weeks, from April to June 2021.

Before actual data collection, we carried out key informant interviews and focus group discussions through which additional information about waste management practices in Lagos and the general perception of the people regarding plastic pollution were obtained. The information obtained helped in updating the questionnaire. The questionnaire was subjected to a pilot study using 30 households selected from three EAs, ten from each. The EAs used for the pilot study were not part of the actual study. The pilot test facilitated the collection of some open-ended questions regarding the people’s opinions on different issues, for example, environmental effects of single-use plastics. The responses to the open-ended questions enabled us to gain insight into the issues and thus helped us provide options regarding those issues in the questionnaire.

### 3.4 Empirical framework or econometric approach and model specification

We first used descriptive statistics to evaluate the respondents’ opinions regarding the waste disposal approach and their perception regarding single-use plastics and their effect on the environment. Secondly, following the conceptual framework, we applied the multinomial logit (MNL) model to estimate the determinants of the waste disposal approach employed by the households to find out the drivers of legal (proper waste disposal) as against illegal disposal. MNL has the advantage of allowing the analysis of decisions involving three or more categories, thereby enabling one to determine probability choices for different categories [51,52]. The categories of waste disposal approach are as follows: dispose of waste in an open waste dump or any space they find around their street/residence; dispose of waste in a bin/dumpster provided by government or government-appointed private sector practitioners (PSPs) in our street/residence; dispose of the waste through informal waste collectors (cart pushers); burn waste generated. The second category, disposal of waste in bin/dumpster provided by the government in our street/residence, considered the legal means of disposal, was used as the base category. The other categories are illegal means of disposal. Respondents selected the major and most frequent way they dispose of their wastes. The MNL has response probabilities given as follows:

$$p_{ij}=\frac{\exp(x_{i}^{\prime }\beta_{j})}{\sum_{l=1}^{m}\exp(x_{i}^{\prime }\beta_{j})},j=1,\ldots ,m$$ (1)

Where $x_{i}^{\prime }$ are the regressors under four groups, namely, household/individual characteristics, voluntary service experience, perception about plastic ban and waste management experiences and with coefficients *β*_*j*_. The model is subject to constraints 0<*p*_*ij*_<1 and $\sum_{l=1}^{m}p_{ij}=1$. Thus, results are interpreted with respect to the base category. The test for independence of irrelevant alternatives (IIA) was done using (Suest-based and regular) Hausman tests. All tests showed that the IIA assumption was satisfied.

Furthermore, a bivariate sample selection model was applied to estimate households’ willingness to participate in a cleanup of road gutters/drainage channels program and the number of man-days/hours they were willing to participate in a week. The bivariate sample selection model [53] has both a participation equation given as:

$$y_{1}\left\{ \begin{array}{c} 1\;if\;y_{1}^{*}>0\\ 0\;if\;y_{1}^{*}\leq 0 \end{array}\right.$$ (2)

and an outcome equation is given as

$$y_{2}\left\{ \begin{array}{c} y_{2}^{*}\;if\;y_{1}^{*}>0\\ -if\;y_{1}^{*}\leq 0 \end{array}\right.$$ (3)

From the model, *y*_2_ is observed when $y_{1}^{*}>0$ while *y*_2_ has no value when $y_{1}^{*}\leq 0$. The standard model is specified as a linear model with error terms for the dormant variables as follows:

$$y_{1}^{*}=x_{1}^{\prime }\beta_{1}+\varepsilon_{1}$$ (4)

$$y_{2}^{*}=x_{2}^{\prime }\beta_{2}+\varepsilon_{2}$$ (5)

Sample selection is confirmed if the correlation between *ε*_1_ and *ε*_2_ is zero.

Specifically, we applied the Heckman sample selection model [54] to estimate the factors that influence households’ willingness to participate in a cleanup of road gutters/drainage channels program (participation equation) before the onset of rains and also estimate the determinants of number of man-days they were willing to participate in a week given their willingness to participate (the outcome equation). The Heckman selection model in line with the bivariate sample selection model is specified as follows:

$$y_{j}=X_{j}\beta +u_{1j}$$ (6)

Regression/Outcome equation

The dependent variable *y*_*j*_ (number of man days or hours a respondent is willing to participate to clean-up road gutters and drainage channels) for observation j is only observed if

$$z_{j}\gamma +u_{2j}>0$$ (7)

Selection equation

Where

$$u_{1}∼N(0,\sigma )$$

$$u_{2}∼N(0,1)$$

$$corr\;(u_{1},u_{2})=\rho$$

Eq 5 (the outcome equation) was estimated using only the respondents who indicated they were willing to participate in cleanup road gutters/drainage channels. Eq 6 (selection equation) was estimated using respondents that were willing to participate (assigned the value of 1) and those not willing to participate (assigned the value of 0) to clean up drainage channels. Some explanatory variables appeared in both Eqs (6) and (7), while some appeared in either of the two equations. In this regard, it is essential to indicate that the explanatory variables’ coefficients included only in the outcome equations are interpreted as marginal effects. In contrast, the coefficients are not interpreted as marginal effects when the variables appear in both equations. When they appear in both equations, the coefficients of the outcome equation are no longer marginal effects because they are influenced by the fact that they were used in the selection equation. However, the marginal effects are derived using the equation:

$$\beta_{k}-(\alpha_{k}*\rho *\sigma_{n}*Dpr)$$ (8)

where, *β* and *α* are coefficient of the variable in Eqs (5) and (6) respectively; ρ (rho) is the correlation coefficient of the error terms in both equations; *σ* (sigma) is the error term in the outcome equation; while Dpr is inverse mills ratio plus the probability of being selected [55].

The model for estimating the determinants of household’s willingness to participate in a cleanup of road-gutters and drainage channels program and the number of man-days they are willing to volunteer in a week is specified as:

$$\begin{array}{l} WTPa_{i}=\\ \beta_{1}+\beta_{2}\mathrm{Hou}\_{\mathrm{size}}_{i}+\beta_{3}\;{\mathrm{Gender}}_{i}+\beta_{4}\mathrm{num}\_\mathrm{years}\_{\mathrm{sch}}_{i}+\\ \beta_{5}\mathrm{expen}\_\mathrm{per}\_{\mathrm{capita}}_{i}+\beta_{6}{\mathrm{married}}_{i}+\beta_{7}{\mathrm{remittances}}_{i}+\\ \beta_{8}\mathrm{sort}\_{\mathrm{waste}}_{i}+\beta_{9}\mathrm{bag}\_{\mathrm{waste}}_{i}+\beta_{10}\mathrm{mem}\_{\mathrm{assoc}}_{i}+\beta_{11}\mathrm{any}\_\mathrm{inc}\_\mathrm{contri}\_{\mathrm{per}}_{i}+\beta_{12}\mathrm{waste}\_\mathrm{mgt}\_{\mathrm{fee}}_{i}+\\ \beta_{13}\mathrm{waste}\_\mathrm{mgt}\_{\mathrm{infoi}}_{i}+\beta_{14}\mathrm{part}\_\mathrm{vol}\_{\mathrm{ser}}_{i}+\\ \beta_{15}\mathrm{invo}\_\mathrm{comu}\_\mathrm{cl}\_{\mathrm{up}}_{i}+\\ \beta_{16}\mathrm{envp}\_\mathrm{blo}\_\mathrm{drain}\_{\mathrm{chan}}_{i} \end{array}$$ (9)

### 3.5 Variables used in the model

The explanatory variable used in the model and their definitions are presented in Table 1. The variables were grouped into household/individual characteristics, social capital, perception about plastic ban and waste management practices. Under household/individual characteristics, we included gender to find whether the gender of a household head explains his/her willingness to volunteer or participate in a cleanup campaign and the number of man-days they are willing to volunteer. We expect that women would be more willing to participate and volunteer more days, given that more women than men are more involved in waste management activities in the household. Women are often environmentally friendlier and are more involved in household waste management activities than men and even work with children and domestic workers to sort and sell recyclables [46,56]. We also included the education of the household head measured as the number of years spent in school. We expect that number of years spent in school will positively influence willingness to participate in the cleanup of gutters and drainage channels to reduce the flow of plastics into the oceans as educated people are knowledgeable about the effect of plastic pollution. Household size was also included, and we expect that household size would positively influence the willingness of households to participate in cleanup campaigns since larger households would have enough people that could be assigned other duties in the household. [46] found that household size was positive and significantly influenced waste separation in Lake Victoria Crescent, Uganda.

**Table 1:** Definition of explanatory variables used in the model.

Variables	Means of Measurement and definitions
**Household/Individual Characteristics**	
Gender of household head	This is a dummy variable. The value is 1 if male; 0 if female
Education level of household head	Measured as total number of years spent in school by the respondent
Another income contributing person in the household	Measured as the total number of other income contributing persons in the household besides the household head
Married	This is a dummy variable. The value is 1 if currently married; 0 if not-married
Received remittances	This is a dummy variable. The value is 1 if the respondent received remittances (from a relative abroad or in another part of Nigeria) in the last 12 months preceding the interview; 0 otherwise.
Household size	Number of persons in a household
**Social Capital**	
Member of an association	This is a dummy variable. The value is 1 if a respondent is a member of a community association or voluntary association; 0 otherwise.
Have participated in voluntary service	This is a dummy variable. The value is 1 if a respondent ever participated in voluntary service in the neighbourhood besides waste cleanup; 0 otherwise.
Involved in community cleanup activities	This is a dummy variable. The value is 1 if a respondent ever participated in a waste cleanup organised by residents of his/her locality; 0 otherwise.
**Perception about plastics ban**	
Don’t ban single-use plastics	This is a dummy variable. The value is 1 if the respondent thinks that plastics should not be banned; 0 otherwise.
**Waste management practices**	
Receive waste management information	This is a dummy variable. The value is 1 if a respondent receives waste management information; 0 otherwise.
Bag waste	This is a dummy variable. The value is 1 if a household bags waste before disposal; 0 otherwise.
Sort waste	This is a dummy variable. The value is 1 if a household sorts waste before disposal; 0 otherwise.
Pay waste management fee	This is a dummy variable. The value is 1 if respondent pays waste management fee; 0 otherwise.
Dumpster	This is a dummy variable. The value is 1 if a household has a waste dumpster located in their vicinity; 0 otherwise.
**Dependent Variables**	
Method of waste disposal	This is a categorical variable with four categories covering four different approaches to waste disposal.
Willingness to participate	This is a dummy variable. The value is 1 if a respondent is willing to participate in a waste cleanup of road gutters and drainage channels program before the onset of rains; 0 otherwise.
Man days/hours per week	Number of man-days the respondent is willing to contribute in a week

In addition, we included some social capital variables in determining the influence of social capital on willingness to volunteer or participate in cleanup campaigns and the number of man-days they are willing to contribute. The variables include membership of an association, participation in voluntary service, and involvement in community cleanup activities. It is expected that household heads with a social capital network, for example, belonging to an association, participating in voluntary service or community cleanup activities, would be willing to participate in cleanup campaigns since they have been involved in related activities in the past. [57] found that higher levels of social capital reduce environmental pollution. Also, we captured the perception of the respondents regarding plastic ban using a variable named “don’t ban single-use plastics”, where those that do not want single-use plastics banned were assigned the value of one, while those that want it banned were assigned the value of zero. The variables under waste management practices include receiving waste management information, bag waste, sorting waste, and paying the management fee. It is expected that respondents who receive waste management information would understand the dangers of improper disposal and the effect of single-use plastic waste on the environment. This knowledge will make them willing to clean up road gutters/drainage channels to reduce the flow of plastics into the ocean. [58] observed that public awareness is a major factor in solid waste management. On the other hand, it is expected that respondent who sort and bag wastes and who pay waste management fees would not be willing to cleanup road gutters/drainage channels. This is because they would feel that they have done what is expected of them by sorting and bagging their waste and paying waste management fees and therefore are not responsible for wastes in road gutters and drainage channels.

We also tested the presence of multi-collinearity among the independent variables by calculating the variance inflation factor (VIF). According to [59], a variable is assumed to be collinear if VIF exceeds 10; thus, variables with VIF above ten were not included in the model.

## 4 Results and discussion

### 4.1. Descriptive statistics of variables used in the model

Table 2 shows the descriptive statistics of variables used in the model The result shows that the mean household size of the respondents was four people, while the mean number of years they spent in school was 12 years. The average amount paid as a waste management fee was 410 naira (amounting to US$0.99 at the current official exchange rate of 416 naira to US$1). The amount paid as a waste management fee varies from location to location and depends on the perceived income level of those residing in the locality.

**Table 2:** Descriptive statistics of variables used in the model.

Variable	Means	Standard Deviation	Minimum	Maximum
**Individual and household characteristics**				
Gender of household head	0.725	0.447	0	1
Education level of household head	12.094	4.760	0	21
Another income contributing person in a household	0.608	0.489	0	1
Married	0.715	0.452	0	1
Received remittances	0.173	0.379	0	1
Household size	3.937	1.680	1	13
**Social Capital**				
Member of an association	0.235	0.424	0	1
Have participated in voluntary service	0.457	0.499	0	1
Involved in community cleanup activities	0.538	0.499	0	1
**Perception about plastics ban**				
Don’t ban single-use plastics	0.788	0.408	0	1
**Waste management practices**				
Receive waste management information	0.540	0.499	0	1
Bag waste	0.867	0.340	0	1
Sort waste	0.197	0.398	0	1
Pay waste management fee	0.713	0.453	0	1
Amount paid as waste management fee	409.855	663.472	0	5000
**Dependent Variables**				
Willingness to clean up drainage channels	0.755	0.430	0	1
Man days/hours per week	1.112	0.616	0.1	6

### 4.2 Perceptions of the respondents regarding the effect of single-use plastics on the environment

**Fig 3:**
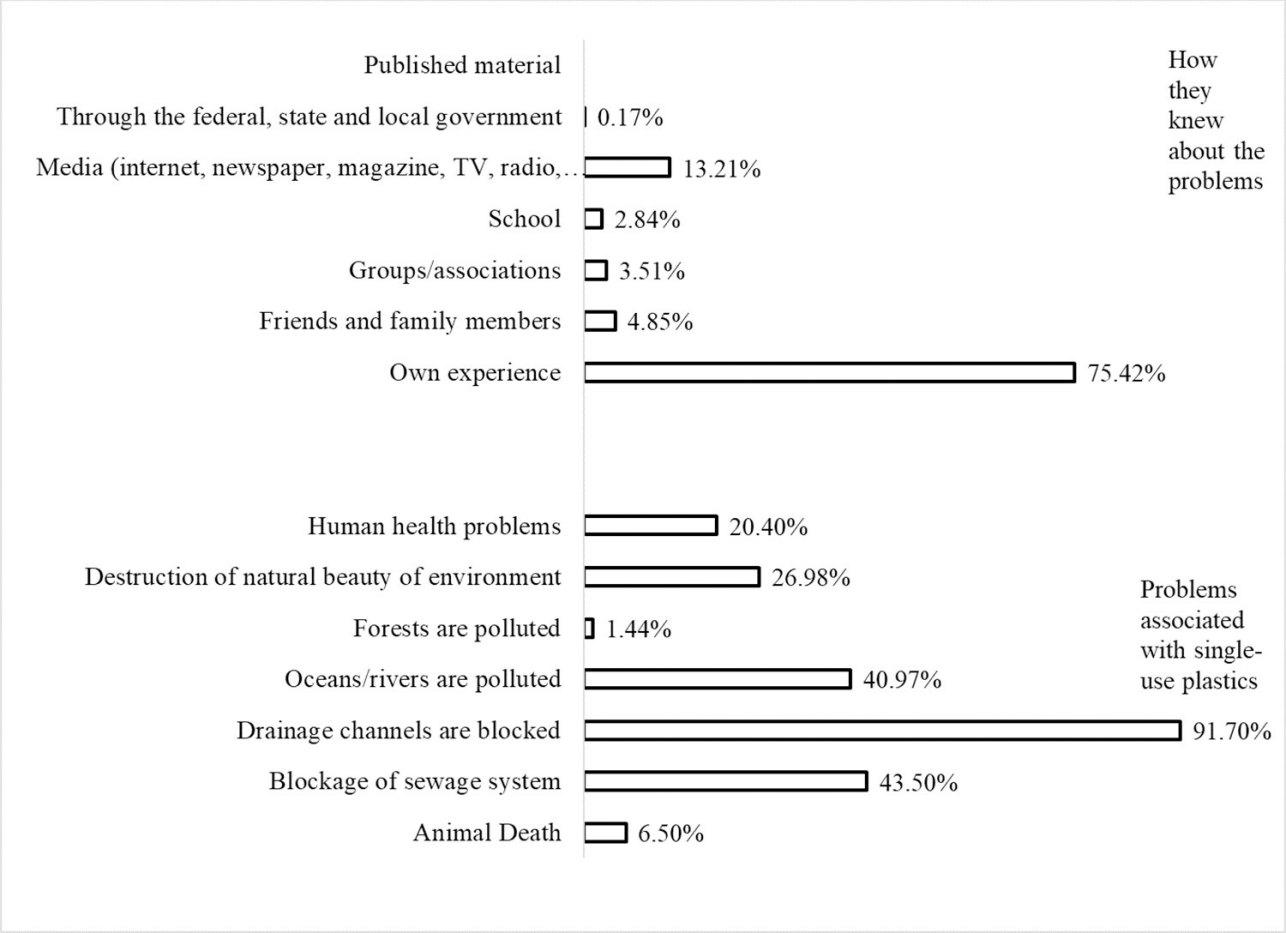
Opinion of the respondents regarding environmental problems associated with single-use plastics and how they knew about it. Given multiple responses, the result shows that most respondents (91.70 percent) indicated that blocking drainage channels was the environmental problem associated with single-use plastics. Also, a high proportion of them believed that blockage of sewage systems (43.50 percent) and pollution of ocean and rivers (40.97 percent) were the problems associated with single-use plastics. Only 1.44 percent of them indicated that forest pollution was one of the problems associated with single-use plastics. This finding suggests that Lagos residents consider blockage of drainage channels, blockage of sewage systems and pollution of oceans and rivers as the main problems associated with single-use plastics.

Regarding the issue of single-use plastics harming the environment, the majority (92.50 percent) believed that single-use plastics harm the environment. Only 6.00 percent indicated that single-use plastics do not harm the environment, while 1.50percent did not know. This finding is in line with that of [60], who found that 97% of interviewed Lagos residents indicated that they are aware of the environmental impacts of using plastics bags. On environmental problems associated with single-use plastics and how they knew about them, the result is presented in Fig 3.

**Fig 4:**
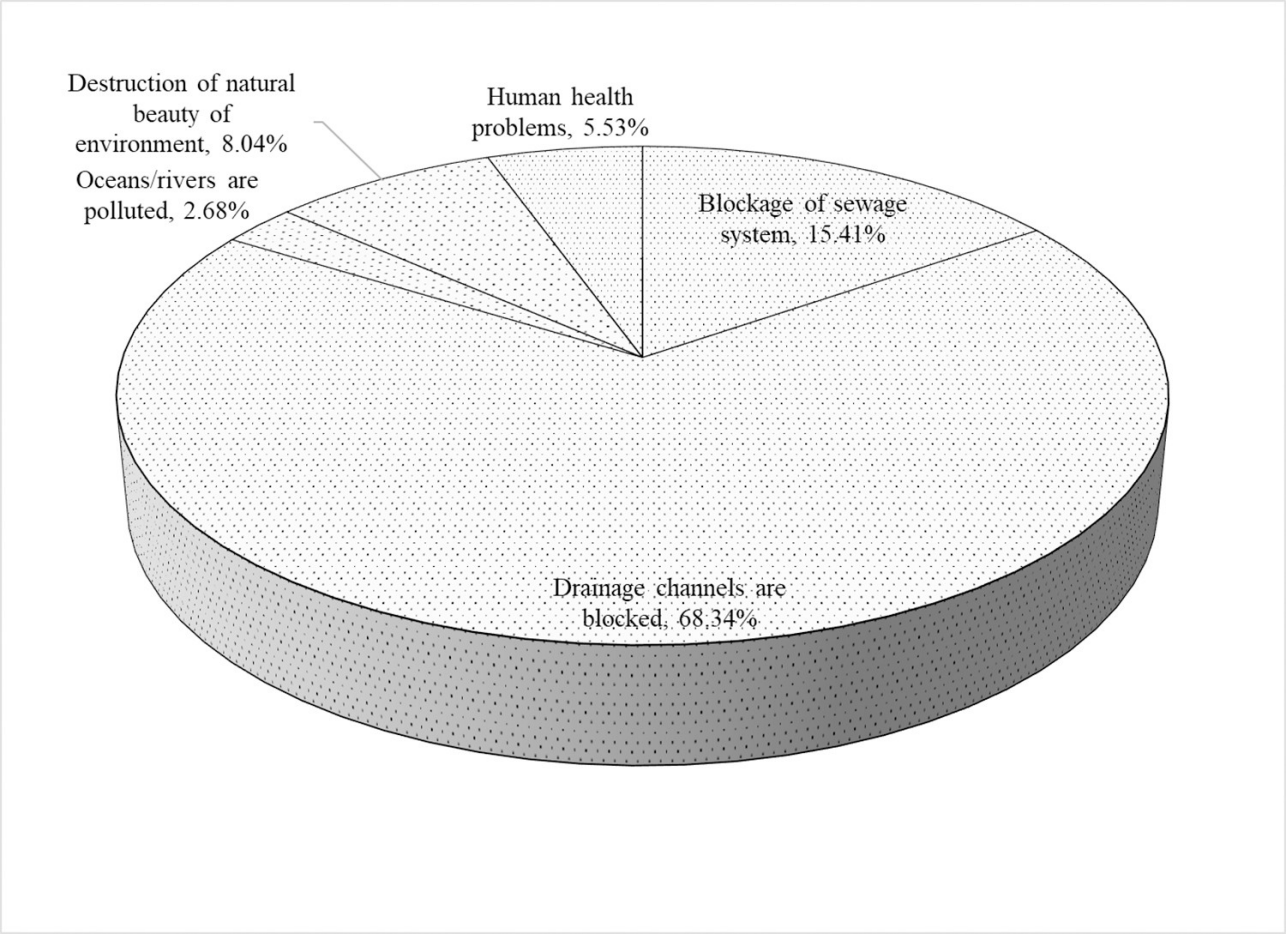
Opinion of the respondents regarding the most severe environmental problems associated with single-use plastics.

On how they knew about the environmental problems associated with single-use plastics, the majority (74.42 percent) indicated that they received the knowledge through their personal experiences. At the same time, none got the knowledge through published material. On the most severe environmental problem associated with single-use plastics, the result presented in Fig 4 shows that the majority (68.34 percent) of the respondents believed blocking drainage channels was the most severe environmental problem caused by single-use plastics. [61] observed that when single-use plastic wastes are improperly disposed of, wind or animals carry them and fill up land spaces and drainage channels. This finding suggests the need to direct some efforts towards cleanup drainage channels to limit the flow of plastics into waterways and oceans, thus justifying the estimation of factors that determine the respondents’ willingness to cleanup drainage channels.

### 4.3 Waste disposal approach employed by the households

On the waste disposal approach employed by the households, the result shows that the highest proportion (47.04 percent) of the respondents dispose of waste through informal waste collectors or cart pushers, 32.58percent dispose of waste in bins/dumpsters provided by government or government-appointed private sector participants (PSPs). In addition, 13.41percent dispose of waste in open waste dumps or any space they found around their street/residence, and 6.97 percent burn the wastes they generate. This result shows that most (67.42 percent) of the households in the coastal city of Lagos dispose of the waste they generate through illegal waste disposal means. This finding aligns with [62] that in many parts of Lagos, residents have adopted illegal and environmentally unfriendly options in disposing of their waste. Our finding suggests that even though most residents are aware of the harmful effects of improper waste disposal and plastic waste, they still practice illegal waste disposal. [63] noted that experience in Japan shows that even though individuals understand the environmental impact of improper waste disposal, they may still dispose of their wastes improperly. Most of the illegally disposed waste, of which a high percentage are plastics, are carried by wind and stormwater through drainage channels into waterways and oceans, as earlier noted. In fact, [64] observed that the first culprit in the pollution of oceans is open dumping or illegal waste disposal.

Furthermore, the result of the MNL regression showing the coefficients and marginal effect of the determinants of the waste disposal approach used by the households is presented in Table 3. The result shows that the chi-square test of the overall specification of the MNL model was highly significant. Following this, we found that some variables significantly reduces the likelihood of illegal disposal in favour of legal disposal (disposal in bins and waste dumpsters). Specifically, the result shows that household size, having ever been involved in community cleanup activities, receipt of waste management information, payment of waste management fee and having a dumpster in locality significantly reduces the likelihood of illegal waste disposal, namely, open dumping of waste, use of informal waste collectors and burning of waste. The result of the marginal effects shows that household size, having ever been involved in community cleanup activities, receive waste management information, payment of waste management fee and having a dumpster in locality reduces the likelihood of open dumping of waste by one percent, 8.4 percent, 17.2 percent, 11.2 percent and 10.7 percent respectively. The result also shows that having ever been involved in community cleanup activities and having a dumpster in a locality reduces the likelihood of waste disposal through informal waste collectors by 11.7 percent and 43.7 percent, respectively. These findings suggest that providing adequate information about waste management and dumpsters in the localities to households, especially targeting those with large households involved in previous community cleanup activities and paying waste management fees, would reduce the illegal disposal of wastes and plastic pollution. Adequate information and communication could be effective as it could help discourage individuals from applying neutralization techniques [63] which they often use to dispel their feelings of guilt regarding illegal waste dumping. The finding regarding the effect of the provision of waste information on facilitating legal waste disposal equally supports the finding of [58] that public awareness was a major factor in solid waste management. Also, [64] noted that providing an effective waste collection and disposal system, such as the provision of dumpsters for waste collection, is crucial for any solution to illegal waste disposal and marine plastic pollution.

**Table 3:** Result of MNL- determinants of waste disposal approach employed by the households.

Variable	Dispose in open waste dump or spaces along a street or near a residence		Dispose of waste through informal waste collectors or cart pushers		Burn waste	
	Coefficients	Marginal Effects	Coefficients	Marginal Effects	Coefficients	Marginal Effects
**Individual and household characteristics**						
Gender of household head	-0.437 (0.436)	-0.007 (0.029)	-0.500* (0.282)	-0.092* (0.054)	-0.038 (0.557)	0.005 (0.007)
Education level of household head	0.0002 (0.039)	-0.0007 (0.002)	0.013 (0.028)	0.003 (0.006)	0.035 (0.047)	0.0004 (0.0007)
Any other income contributing person in a household	0.606 (0.420)	0.0006 (0.027)	0.920*** (0.279)	0.188*** (0.057)	0.152 (0.565)	-0.008 (0.010)
Married	0.212 (0.473)	0.038 (0.026)	-0.480 (0.316)	-0.122** (0.060)	0.145 (0.595)	0.006 (0.008)
Received remittances	-0.843 (0.595)	-0.049* (0.028)	-0.076 (0.307)	0.020 (0.065)	-0.369 (0.631)	-0.004 (0.008)
Household size	-0.301** (0.118)	-0.014* (0.008)	-0.166** (0.079)	-0.019 (0.016)	-0.458*** (0.156)	-0.005* (0.003)
**Voluntary service experience**						
Have participated in voluntary service	0.988** (0.433)	0.010 (0.027)	1.219*** (0.281)	0.211*** (0.053)	1.857*** (0.575)	0.017 (0.011)
Ever involved in community clean-up activities	-0.995** (0.415)	-0.084*** (0.032)	0.149 (0.266)	0.117** (0.054)	-2.227*** (0.603)	-0.046** (0.021)
**Perception about plastic ban**						
Don’t ban single-use plastics	0.970** (0.500)	0.048** (0.024)	0.332 (0.289)	0.037 (0.063)	0.231 (0.602)	-0.0007 (0.009)
**Waste management practices**						
Receive waste management information	-2.700*** (0.422)	-0.172*** (0.034)	-0.977*** (0.255)	-0.060 (0.052)	-1.261** (0.509)	-0.006 (0.008)
Pay waste management fee	-1.955*** (0.425)	-0.112*** (0.040)	-0.786** (0.320)	0.045 (0.060)	-4.015*** (0.656)	-0.135*** (0.045)
Dumpster in locality	-3.424*** (0.486)	-0.107*** (0.025)	-2.670*** (0.280)	-0.437*** (0.049)	-3.579*** (0.835)	-0.023** (0.011)
Constant	3.312*** (0.887)		2.617*** (0.646)		3.441*** (1.022)	
LR chi2 (36)	433.18					
Prob > chi2	0.000					
Log likelihood	-458.001					
Total number of observation	600					

**Base category**: Dispose of in bins/dumpsters provided by the government in our neighbourhood (legal disposal).

Note: *, ** and *** indicates significance at p<0.10, p<0.05 and p<0.01, respectively; variables in brackets are standard errors.

Source: Calculated from field survey data, 2021.

On the other hand, the result shows that having the perception that single-use plastics should not be banned increases the likelihood of open dumping of waste. This implies that those who said that plastics should not be banned also carry out illegal waste dumping. The marginal effect results show that having the perception that plastics should not be banned increases the likelihood of open dumping of waste by 5 percent. This finding suggests the need to provide information on the dangers of single-use plastics to households to discourage illegal dumping of wastes. Also, although not expected, the result equally shows that participating in voluntary service increases the likelihood of illegal waste disposal, that is, open dumping of waste, use of informal waste collectors and burning of waste. The marginal effects show that participating in voluntary service increases the likelihood of waste disposal through informal waste collectors and cart pushers by 21 percent. The high marginal effect could be because those who participated in voluntary service saw the informal waste collectors as “voluntary people” who filled the space and helped in providing a service that the government did not provide. Besides, research evidence [63] shows that people aware of the dangers of illegal dumping of wastes still practice it.

### 4.4 Willingness of the respondents to clean-up drainage channels and number of man-days they are willing to contribute

**Fig 5:**
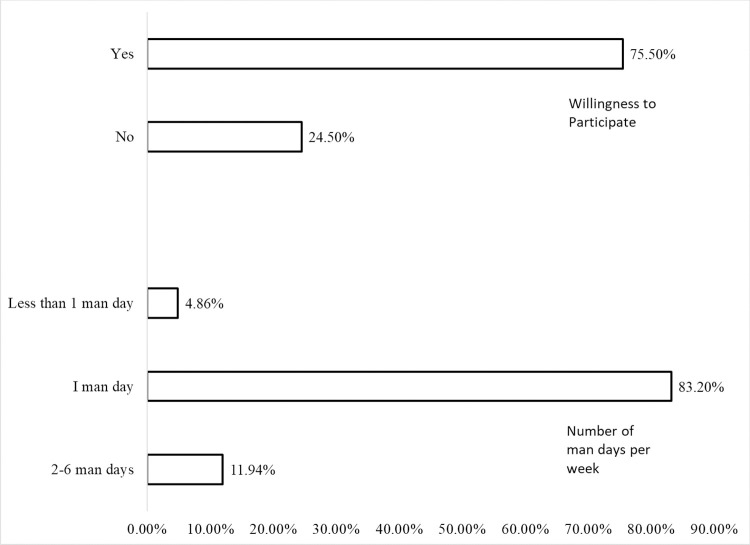
Result of the willingness of the respondents to participate in a cleanup of road gutters/drainage channels program and the number of man-days they are willing to contribute.

The result of the household heads’ willingness to participate in a program for the cleanup drainage channels is presented in Fig 5. The result shows that the majority (75.5 percent) of the respondents were willing to participate in the cleanup of road gutters/drainage channels before the onset of rains to reduce the flow of plastics into the ocean. We also found that most of the respondents, 83.20 percent, indicated that they would contribute only one man-day (8 hours) per week. This finding suggests that the households would be involved in the cleanup drainage channels if mobilized; however, they would contribute only one man-day in a week in the activity. This finding supports [36] that about 74.1percent of the respondents in China’s Zhejiang province were willing to participate in beach cleanup program and are also willing to give up 1.5 days per month on average.

### 4.5 Determinants of willingness of the respondents to participate in the clean-up road gutter/drainage channels and number of man-days they are willing to contribute

The result of the sample selection model of the determinants of the respondents’ willingness to clean up drainage channels and the number of man-days they are willing to contribute is presented in Table 4. The result shows that the rho (ρ) was significantly different from zero thus, justifying the use of a Heckman sample selection model. Also, the result shows that some variables in the selection and outcome equations significantly influenced the willingness of the respondents to participate in a programme for the clean-up of road gutters and drainage channels and the number of man-days they are willing to participate in a week. Specifically, the gender of the household head significantly reduces the likelihood of willingness to participate in the clean-up of drainage channels and equally the number of days they are willing to participate or volunteer. Given that gender is a dummy variable, where the value is 1 if male; 0 if female, it suggests that being a male household head reduces the likelihood of willingness to participate in the clean-up of drainage channels and the number of man-days they are willing to contribute/volunteer. Thus, females are more willing to participate and contribute more man-days to clean road gutters/drainage channels than men. Considering that gender appeared both in the selection and outcome equations, the parameters are not the marginal effects. Thus using Eq (8) above, the mean (-0.152) of the corrected coefficient is shown in Table 5. Thus being a male reduces the likelihood of a household head contributing man-days to cleanup road-gutters/drainage channels by 15 percent. The findings regarding the willingness of females to participate and contribute more number of man-days is expected as women are more involved in waste management in households than men. As noted by [56], Women are environmentally friendlier and are more involved in household waste management activities than men. This finding aligns with [35] that female visitors were more willing to participate and give up more days than male visitors in a beach cleanup scheme. This finding also supports [46,65] that gender significantly reduced the likelihood of involvement in waste separation, with men being less involved in waste separation than women. The result equally shows that the household head’s education (number of years spent in school) significantly reduces the likelihood of his/her contribution of days to the cleanup of road gutters and drainage channels. This finding suggests that the higher the number of years a household head spent in school, the lower the number of days he/she would contribute to the cleanup activities to reduce the flow of plastics into the ocean. This could be because those with more education are more engaged in other jobs and employment and may have little time contributing to drainage cleanup activities to reduce the flow of plastics into the oceans. This finding is in line with [65] that those with low levels of education were more likely to separate waste than those with higher education. We also found that having additional income contributing members in a household increases the likelihood of participation in cleanup of drainage channels but does not influence the number of man-days they are willing to contribute. A household having an additional income contributing person suggests that the household has more income than a household that does not have. [35] also found that higher-income respondents were willing to participate in beach cleanup than low-income respondents.

**Table 4:** Result of Heckman selection model showing the determinants of the willingness of the respondents to participate in the cleanup drainage channels and number of man-days they are willing to contribute.

Variable	Selection equation results (willingness to participate)	Outcome equation results (number of days to volunteer)
**Individual and household characteristics**		
Gender of household head	-0.296** (0.141)	-0.229*** (0.072)
Education level of household head	-0.001 (0.012)	-0.014** (0.007)
Any other income contributing person in a household	0.152 (0.132)	0.235*** (0.070)
Married	0.064 (0.142)	-0.006 (0.077)
Received remittances		0.066 (0.067)
Household size	-0.030 (0.037)	-0.045** (0.019)
**Social Capital**		
Member of an association	-0.278 (0.200)	0.042 (0.086)
Have participated in voluntary service	0.923*** (0.174)	0.279*** (0.078)
Ever involved in community clean-up activities	0.357*** (0.126)	0.107 (0.070)
**Perception about plastics ban**		
Don’t ban single-use plastics	-0.033 (0.141)	0.049 (0.077)
**Waste management practices**		
Receive waste management information	-0.005 (0.123)	0.166** (0.066)
Bag waste	0.090 (0.164)	-0.070 (0.091)
Sort waste	0.118 (0.156)	0.035 (0.082)
Pay waste management fees	0.203** (0.105)	
Amount paid as waste management fees		-0.0001** (0.00005)
Constant	0.078 (0.275)	1.004*** (0.152)
Rho (ρ)	0.936*** (0.014)	
Wald (chi2(14)	83.30	
Prob > chi2	0.000	
Log likelihood	-632.234	
Total number of observation	600	
Selected Observations	453	
Non selected Observations	147	
LR test if independent equations (rho = 0): Chi2(1)	102.08***	

Note: *, ** and *** indicates significance at p<0.10, p<0.05 and p<0.01, respectively.

Source: Calculated from field survey data, 2021.

**Table 5:** Mean and standard deviation of corrected coefficients for significant variables in outcome equation that also appeared in the selection equation.

Variable	Observations	Mean	Standard Deviation	Minimum	Maximum
Real Gender	600	-0.152	0.051	-0.223	-0.025
Have participated in voluntary service	600	0.039	0.160	-0.360	0.258

Source: Computation from field survey data 2021.

Note: Corrected coefficients are the marginal effect of the variables.

In addition, social capital variables which include, having participated in voluntary service and having ever been involved in community cleanup activities, significantly influenced the willingness of households to participate in the cleanup of drainage channels program. The finding also shows that participating in voluntary service significantly increases both the willingness of a household head to participate and the number of days they are willing to contribute. Given that the variable appeared in both the selection and outcome equation, the result of mean correction shows that participation in voluntary services significantly increases the likelihood of contribution of man-days to the cleanup of road-gutters and drainage channels by four percent (Table 5). Also, having been involved in community cleanup activities significantly increases the likelihood of participation in the clean-up of road gutters/drainage channels program but did not influence the number of days they were willing to contribute. These findings are expected given that those used to participating in a given activity would quickly get involved in such activity. This finding suggests that social capital increases the likelihood of involvement in cleanup campaigns. This finding supports [66] that a community’s social capital was significantly correlated with its recycling performance. Also, the study of [57] revealed that higher levels of social capital reduced environmental pollution.

The result equally shows that the waste management practice of the households influenced their willingness to participate in the cleanup of drainage channels and the number of man days they are willing to contribute. Specifically, the result shows that paying waste management fees increased significantly the likelihood of a household’s participation in the clean-up of road gutters and drainage channels program to reduce the flow of plastic into the ocean. On the other hand, the number of days the households are willing to contribute reduces with an increase in the amount they pay for waste management. This suggests that a household that pays a waste management fee would be willing to clean up drainage channels to reduce the flow of plastics into the ocean. However, the households that pay higher fees would not contribute many days to the clean-up of road gutters and drainage channels program. This finding is expected as those that pay waste management fees are already committed to waste management and would be willing to participate in waste management activities regarding plastic pollution. However, those that pay more may feel that the money they pay could account for the contribution of days and would not be willing to contribute many days. This finding is in line with that of [45] that found that willingness to pay for waste disposal and paying for waste disposal positively and significantly influenced households’ involvement in waste separation for recycling. In addition, the result shows that the receipt of waste management information significantly increases the number of days the households are willing to contribute to the cleanup of road gutters/drainage channels to reduce the flow of plastics into the ocean. This finding is expected as those that receive information would have some knowledge regarding the effect of plastic waste on the environment. This finding supports the observation of that public awareness was a significant factor in solid waste management.

## 5 Conclusions and recommendations

The study estimated the determinants of households’ waste disposal approach, their willingness to participate and the number of man days they are willing to contribute to a clean-up of road gutters and drainage channels program to limit the flow of plastics into the ocean. The study found that most (67.42 percent) of the households in the coastal city of Lagos dispose of the waste they generate illegally. Some variables, namely, household size, having ever been involved in community cleanup activities, receipt of waste management information, payment of waste management fee and having a dumpster in a locality, significantly reduced the likelihood of illegal waste disposal. Also, most (75.50 percent) of the households were willing to clean up road gutters/drainage channels before the onset of rains to reduce the flow of plastics into the oceans. However, the majority (83.20 percent) were only willing to contribute one day in the week to carry out the activity. The study also found that some explanatory variables, namely, gender of the household head and participation in voluntary service, significantly influenced both households’ willingness to participate and the number of man-days they were willing to contribute to reducing the flow of plastics into the oceans. On the other hand, some explanatory influenced either household’s willingness to participate or the number of man-days they were willing to contribute to reducing the flow of plastics into the oceans. Generally, our study has provided evidence-based information that could help manage the problem of illegal waste disposal and reduce the flow of plastics into the oceans.

Following the findings, we can infer that households in Lagos practice illegal waste disposal. However, most households are willing to participate in a clean-up of road gutters/drainage channels programme once a week before the onset of seasonal rains to reduce the flow of plastics into the oceans. Interestingly, the provision of waste management information and dumpsters reduces the likelihood of illegal waste disposal. Also, women and those who often participate in volunteer activities are more likely to participate and contribute more days in the cleanup activities. In addition, large households with highly educated household heads and those that pay more for waste management would contribute fewer days in the cleanup activities. However, enhancing the knowledge of the household heads on the dangers of plastic pollution through well-packaged information on waste management would help increase the number of man-days they can contribute to cleanup activities to reduce the flow of plastics into the oceans. Therefore, to reduce the flow of plastics into the oceans, the government at the local level should make an effort to provide waste dumpsters and mobilize the citizens to clean up road gutters/drainage channels before the onset of rains. Government should provide well-packaged information on the dangers of single-use plastics and plastic pollution to the citizens, especially for the men folks, to enhance their knowledge on the issue and thus discourage illegal waste disposal. It will also encourage them to take action against plastic pollution. Mobilization of efforts for a clean-up campaign should focus mainly on previous volunteers to community clean-up activities, women, less educated household heads, households with additional income contributing members, low waste management fee-paying households. Incorporating these findings in policy and actions against plastic pollution would help reduce the flow of plastics into the ocean.

## Supporting information

S1 File (PDF)

## References

[1] Wilcox C, Mallos NJ, Leonard GH, Rodriguez A, Hardesty BD. Using expert elicitation to estimate the impact of plastic pollution on marine wildlife. Mar Pol. 2016; 65: 107–114. 10.1016/j.marpol.2015.10.014.

[2] Jambeck JR, Geyer R, Wilcox C, Siegler TR, Perryman M, Andrady A, et al. Plastic waste inputs from land into the ocean. Sci. 2015; 347: 768–771. doi: 10.1126/science.1260352 25678662

[3] Bakare W. Solid waste management in Nigeria 2021. Available from: https://www.bioenergyconsult.com/solid-waste-nigeria/.

[4] Adeyemo O. Integrated waste management initiative of the local government: a case study of Lagos, Nigeria. Lagos State Waste Management Authority 2019. Available from: https://wedocs.unep.org/bitstream/handle/20.500.11822/33699/1/IWMI.pdf.

[5] Alpizar F, Carlsson F, Lanza G, Carney B, Daniels RC, Jaime M, et al. A framework for selecting and designing policies to reduce marine plastic pollution in developing countries. Environ Sci Pol. 2020; 109: 25–35. doi: 10.1016/j.envsci.2020.04.007

[6] UNEP. Marine Plastic Debris and Microplastics—Global Lessons and Research to Inspire Action and Guide Policy Change. Nairobi: United Nations Environment Programme; 2016.

[7] Macfadyen G, Huntington T, Cappell R. Abandoned, lost or otherwise discarded fishing gear. FAO Fisheries and Aquaculture Technical Paper, 523, 2009; 185: UNEP regional seas reports and studies.

[8] Jambeck J, Hardesty BD, Brooks AL, Friend T, Teleki K, Fabres J, et al. Challenges and emerging solutions to the land-based plastic waste issue in Africa. Mar Pol. 2017; doi: 10.1016/j.marpol.2017.10.041

[9] Vegter AC, Barletta M, Beck C, Borrero J, Burton H, Campbell ML, et al. Global research priorities to mitigate plastic pollution impacts on marine wildlife. End Spec Res. 2014; 22: 225–247.

[10] Mouat J, Lozano RL, Bateson H. Economic Impacts of Marine Litter 2010; Kommunenes Internasjonale Miljøorganisasjon (KIMO).

[11] Golden CD, Allison EH, Cheung WWL, Dey MM, Smith M, et al. Nutrition: Fall in fish catch threatens human health. Nat. 2016; 534: 317–320. doi: 10.1038/534317a 27306172

[12] Beaumont NJ, Aanesen M, Austen MC, Börger T, Clark JR, Cole M, et al. Global ecological, social and economic impacts of marine plastic. Mar Pol Bul. 2019; 142: 189–195. doi: 10.1016/j.marpolbul.2019.03.022 31232294

[13] Willis K, Maureaud C, Wilcox C, Hardesty BD. How successful are waste abatement campaigns and government policies at reducing plastic waste into the marine environment? Mar Pol. 2018. doi: 10.1016/j.marpol.2017.11.037

[14] Federal Republic of Nigeria. National Policy on Plastic Waste Management. Abuja: Federal Ministry of Environment; 2020. doi: 10.1016/j.wasman.2020.10.019

[15] Nwafor N, Walker TR. Plastic Bags Prohibition Bill: A developing story of crass legalism aiming to reduce plastic marine pollution in Nigeria. Mar Pol. 2020; 120: 104160. doi: 10.1016/j.marpol.2020.104160

[16] Gamaralalage PJD, Onogawa K. Strategies to Reduce Marine Plastic Pollution from Land-based Sources in Low and Middle—Income Countries. Nairobi: United Nations Environment Program; 2019. doi: 10.1016/j.jhazmat.2018.07.023

[17] Xanthos D, Walker TR. International policies to reduce plastic marine pollution from single-use plastics (plastic bags and microbeads): A review. Mar Pol Bul. 2017; 118(1–2): 17–26. doi: 10.1016/j.marpolbul.2017.02.048 28238328

[18] Behuria P. Ban the (plastic) bag? Explaining variation in the implementation of plastic bag bans in Rwanda, Kenya and Uganda. Environment and Planning C: Politics and Space. 2021; 239965442199483. doi: 10.1177/2399654421994836

[19] Heinrich Böll Foundation. Plastic Atlas: Facts and Figures about the World of Synthetic Polymers. Nigeria Edition; 2020.

[20] Royer SJ, Ferrón S, Wilson ST, and Karl DM. (2018) Production of methane and ethylene from plastic in the environment. PLoS ONE 2018; 13: 1–13. doi: 10.1371/journal.pone.0200574 30067755 PMC6070199

[21] Hardesty BD, Good TP, Wilcox C. (2015) Novel methods, new results and science-based solutions to tackle marine debris impacts on wildlife. Ocean Coast. Mana. 2015; 115: 4–9.

[22] UNEP. Valuing Plastics: The Business Case for Measuring, Managing and Disclosing Plastic Use in the Consumer Goods Industry. Nairobi: UNEP; 2014.

[23] Pilz H, Brandt B, and Fehringer R. The impact of plastics on life cycle energy consumption and greenhouse gas emissions in Europe: Summary Report. Vienna: denkstatt GmbH Hietzinger Hauptstraße 28.1130; 2010.

[24] McManamon C. Ban on plastics could increase damage to earth; 2018. Available from: https/www.hw.ac.uk/news/articles/2018/a-plastic-bancould-increase-damage-to.htm.

[25] Trucost. Plastics and sustainability: a valuation of environmental benefits, costs and opportunities for continuous improvement; 2016. Available from: www.trucost.com.

[26] Gupta K. Consumer Responses to Incentive to Reduce Plastic Bag Use: Evidence from a Field Experiment in Urban India. Working Papers 65–11: The South Asian Network for Development and Environmental Economics (SANDEE); 2011.

[27] Ocean Conservancy. International Coastal Cleanup; 2021. Available from: https://oceanconservancy.org/trash-free-seas/international-coastal-cleanup/.

[28] Keep America Beautiful. 2021. Keep America Beautiful; 2021. Available from: https://www.kab.org/.

[29] UN Environment. The clean seas campaign on marine litter; 2017. Available from: www.cleanseas@un.org.

[30] Ezeah C, Roberts CL. Analysis of barriers and success factors affecting the adoption of sustainable management of municipal solid waste in Nigeria. J Environ Mana. 2012; 103: 9–14. doi: 10.1016/j.jenvman.2012.02.027 22459066

[31] Channels Television. Lagos State stops monthly environmental sanitation; 2016. Available from: https://www.channelstv.com/2016/11/23/lagos-state-stops-monthly-environmental-sanitation/.

[32] Piñeiro V, Arias J, Dürr J, Elverdin P, Ibáñez MA, Kinengyere A, et al. A scoping review on incentives for adoption of sustainable agricultural practices and their outcomes. Nat Sust. 2020; 3: 809–820, 10.1038/s41893-020-00617-y.

[33] Hartley BL, Thompson RC, Pahl S. Marine litter education boosts children’s understanding and self-reported actions. Mar Pol Bul. 2015; 90: 209–217. doi: 10.1016/j.marpolbul.2014.10.049 25467869

[34] Afroz R, Rahman A, Masud MM, Akhtar R. The knowledge, awareness, attitude and motivational analysis of plastic waste and household perspective in Malaysia. Environ Sci Pollu Rea. 2017; 24: 2304–2315. doi: 10.1007/s11356-016-7942-0 27812970

[35] Brouwer R, Hadzhiyska D, Ioakeimidis C, Ouderdorp H. The social costs of marine litter along European coasts. Ocea Coas Man. 2017; 138: 38–49. doi: 10.1016/j.ocecoaman.2017.01.011

[36] Manhong S, Mao D, Xie H, LI C. The social costs of marine litter along the East China sea: evidence from ten coastal scenic spots of Zhejiang province China. Sust. 2019; 11: 1807, doi: 10.3390/su11061807

[37] Plastics Europe. Plastics—The Facts 2014/2015: An Analysis of European Plastics Production, Demand and Waste Data. Plastics Europe: Association of Plastic Manufacturers; 2015.

[38] Löhr A, Savelli H, Beunen R, Kalz M, Ragas A, Van Belleghem F. Solutions for global marine litter pollution. Cur Op Environ Sust. 2017; 28: 90–99. 10.1016/j.cosust.2017.08.009.

[39] Aziegbe FI. Seasonality and environmental impact status of polyethylene (cellophane) generation and disposal in Benin City, Nigeria. J Hum Eco. 2007; 22: 141–147.

[40] Murdock H. Environmentalists: drinking water bags harming Nigeria; 2013. Available from: https://www.voanews.com/a/environmentalists-say-drinkingwater-bags-harmng-nigeria/1647923.html.

[41] Omole DO, Ndambuki JM, Balogun K. Consumption of sachet water in Nigeria: quality, public health and economic perspectives. Afr J Sci Tech Inno Dev. 2015; 7: 45–51.

[42] Dumbili E, Henderson L. The challenge of plastic pollution in Nigeria. Plastic Waste and Recycling. 2020; 569–583. doi: 10.1016/b978-0-12-817880-5.00022–0

[43] Kokkinos K, Karayannis V, Lakioti E, Moustakas K. Exploring social determinants of municipal solid waste management: survey processing with fuzzy logic and self-organised maps. Environ Sci Pol Res. 2019; doi: 10.1007/s11356-019-05506-2 31140085

[44] Monella J, Leyaro V. Determinants of Households Willingness to Participate in Solid Waste Separation for Reduce, Reuse and Recycle: The Case of Dar es Salaam. Tanz Econ Rev. 2017; 3: 57–82.

[45] Oyekale AS. Determinants of households’ involvement in waste separation and collection for recycling in South Africa. Environ Dev Sust. 2018; 20: 2343–2371. 10.1007/s10668-017-9993-x.

[46] Ekere W, Mugisha J, Drake L. Factors influencing waste separation and utilisation among households in the Lake Victoria crescent, Uganda. Waste Mana. 2009; 29: 3047–3051. doi: 10.1016/j.wasman.2009.08.001 19740642

[47] Wang F, Cheng Z, Reisner A, Liu Y. Compliance with household solid waste management in rural villages in developing countries. J Cle Prod. 2018; 202: 293–298. doi: 10.1016/j.jclepro.2018.08.135

[48] Momoh JJ, Oladebeye DH. Assessment of awareness, attitude and willingness of people to participate in household solid waste recycling program in Ado-Ekiti, Nigeria. J App Sci Environ Sanit. 2010; 5: 93–105.

[49] FGN. Federal Government of Nigeria Official Gazette: Legal Notice of the Publication of the 2006 Census. Lagos: National Population Commission; 2007.

[50] World Bank, Survey Solutions CAPI/CAWI platform. Washington DC: The World Bank; 2016.

[51] Madalla G. Limited dependent and qualitative variables in econometrics. Cambridge: Cambridge University Press; 1983.

[52] Wooldridge JM. Econometric analysis of cross section and panel data. Cambridge: MIT Press; 2002.

[53] Cameron AC, Trivedi PK. Microeconometric methods and application. Cambridge: Cambridge University Press; 2005.

[54] Heckman JJ. Sample selection bias as a specification error. Econ. 1979; 47: 153–161.

[55] Sweeney K. 2003. Implementing and Interpreting Sample Selection Models. Political Research Lab. Department of Political Science, Ohio State University. 2003; Available from https://polisci.osu.edu/sites/polisci.osu.edu/files/selection_models.pdf.

[56] Hayes B.C., 2001. Gender, scientific knowledge, and attitudes toward the environment. A cross-national analysis. Pol. Res. Quar. 54, 657–671.

[57] Wang Y, Xiong J, Li W, Na M, Yao M. The effect of social capital on environmental pollution in China- suppression or promotion. Inter J Environ Res Pub Heal. 2020; 17: 9459; doi: 10.3390/ijerph17249459 33348783 PMC7766933

[58] Abdel-Shafy H I, Mansour MSM. Solid waste issue: Sources, composition, disposal, recycling, and valorisation. Egyp J Pet. 2018; doi: 10.1016/j.ejpe.2018.07.003

[59] Greene HW. Econometric Analysis, 8th ed. New Jersey: Pearson Education; 2018.

[60] Aligbe MO, Investigating the use of plastic bags in Lagos, Nigeria. Master thesis in Sustainable Development at Uppsala University. 2021; No. 2021/8: 1–96, 30 ECTS/hp.

[61] Kehinde O, Ramonu OJ, Babaremu KO, Justin LD. Plastic wastes: environmental hazard and instrument for wealth creation in Nigeria. Heli. 2020; 6: e05131. doi: 10.1016/j.heliyon.2020.e05131 33024850 PMC7530290

[62] Olafioye O. Refuse disposal: Lagosians embrace dangerous options. The Sun. 2021 Nov 28 [Cited 2022 February 13]. Available from: https://www.sunnewsonline.com/refuse-disposal-lagosians-embrace-dangerous-options/.

[63] Chu AMY. Illegal Waste Dumping under a Municipal Solid Waste Charging Scheme: Application of the Neutralization Theory. Sustain. 2021; 13: 9279. 10.3390/su13169279.

[64] Navarro Ferronato N, and Torretta V. Waste Mismanagement in Developing Countries: A Review of Global Issues. Int. J. Environ. Res. Pub. Heal. 2019; 16: 1060. doi: 10.3390/ijerph16061060 30909625 PMC6466021

[65] Banga M. Household Knowledge, Attitudes and Practices in Solid Waste Segregation and Recycling: The Case of Urban Kampala. Zamb Soc Sci J. 2011; 2: 27–39.

[66] Tsai T. The impact of social capital on regional waste recycling. Sust Dev. 2008; 16: 44–55. doi: 10.1002/sd.326

