# Supplementary material for: Determinants of household’s waste disposal practices and willingness to participate in reducing the flow of plastics into the ocean: Evidence from coastal city of Lagos Nigeria
Source: PLoS One. 2022 Apr 28;17(4):e0267739. doi: 10.1371/journal.pone.0267739 (PMC9049445; doi:10.1371/journal.pone.0267739)
Supplement: S1 File — (PDF) [file pone.0267739.s001.pdf]

THE RESEARCH DIRECTORATE
UNIVERSITY OF NIGERIA, NSUKKA
Director: Professor Obinna E. Onwujekwe *MBBS, MSc, CHM, PhD, DLSHTM;*
Phone: +234 803 700 7771 **E-mail:** obinna.onwujekwe@unn.edu.ng

March 8 2021

Dr Nnaemeka Chukwuone
Department of Agricultural Economics
University of Nigeria Nsukka

RE: REQUEST FOR APPROVAL OF THE STUDY PROTOCOL BY THE RESEARCH DIRECTORATE

Your request regarding the approval of your study protocol for the study titled “Plastic Ban and Plastic Waste Management: Perceptions, Preferences and Willingness to Pay by Residents of Lagos Nigeria” refers.

This is to convey the approval of the Research Directorate of the University of Nigeria Nsukka, for your study to proceed. The directorate has assessed and found that your study has no ethical issues and will not cause any harm to the study participants. Hence, you should proceed with your study and ensure that obtain the signed informed consents of the respondents before conducting the interviews.

Yours faithfully,

Prof Obinna Onwjekwe
